# The Present State of Our Knowledge in Regard to the Sensitiveness of Dentine, and Its Treatment

**Published:** 1902-07

**Authors:** William H. Potter


### Reviews of Dental Literature.

The Present State of our Knowledge in regard to the Sensitiveness of Dentine, and its Treatment. By Professor Dr. Walkhoff, Munich.[1]

In discussing the cause for the sensitiveness of dentine the author declares emphatically against the presence of nerve-fibres in the tubuli of the dentine. He spends considerable space in refuting the views of Morgenstern, who claims to have found nerve-branches in the contents of the tubuli. He agrees with Gysi in that the latter denies the presence of nerve-branches in the tubuli, but disagrees with him in his explanation of the way in which dentine becomes sensitive, Gysi's view being that the contents of the tubuli being semifluid, a pressure or pull upon the contents of the tubuli was immediately transmitted to the odontoblastic cells, and they in turn acted upon the nerve-fibres which surrounded the cells.

---

[1] Der augenblickliche Stand der Kentniss und der Behandlung des sensiblen Dentins, von Hofzahnarzt Prof. Dr. Walkhoff. München. Deutsche Monatsschrift für Zahnheilkunde, 17. Januar, 1902.

Gysi's theory can be called the hydrostatic theory, and does not grant the contents of the tubuli any further share in the establishment of sensitiveness in dentine except as the contents of the tubuli act as confined columns of water and transmit impressions to the odontoblastic cells. The author's views upon this subject can best be understood by the translation of a few sentences. "My view is that we must consider the odontoblastic cell and its projection into the dentinal tubule as physiologically one. . . . The projections into the dentinal tubules are an integral part of those cells which by projections are related to other cells of the pulp. These cells are surrounded by numerous actual nerve-fibres, which in some cases enter between the odontoblasts." "The zeal with which the presence of nerve-fibres in dentine is sought for by many observers is due to the old idea that the condition called irritability is limited to the nerves of an organism. Physiology has long ago overthrown such a view. Irritability, as Verworn has said, is a common peculiarity possessed by all living substance. The tissues of animals which possess no nervous system, and, further, all plant tissue, and even all isolated one-celled organisms react in an outspoken and clear way to all irritation which is brought to bear upon them."

From these considerations the author argues that the contents of the dentinal tubules are capable of receiving an impulse or an irritation and transmitting it to the odontoblastic cells of which they are merely projections, and that the odontoblastic cells are capable of sending on the impulse to the nerve-fibres which immediately surround the cells. And all this is accomplished not by the presence of nerve-fibres in the tubuli, but by the power which all protoplasm has of reacting to an outside stimulus.

The author next considers the clinical diagnosis between normal and hypersensitive dentine. His method is to force by means of a syringe ten or twelve drops of water of a given temperature into a cavity and note the reaction of the dentine. According to his experiments normal dentine reacts at a temperature of 15° to 18° C. That is, a distinct sensation is experienced when water of this temperature is introduced into a cavity. The dentine which reacts at a temperature of 18° to 23° C. is hypersensitive. In this way we have a sure objective means of distinguishing between a purely local affection of the odontoblasts and their projections into the dentinal tubules, and affections of the pulp, especially the acute form of inflammation. An acute inflammation of the pulp reacts powerfully and continuously at a temperature of 23° to 28° C. A pure hyperæsthesia or a normal dentine does not commonly react at this temperature. As to the practical handling of sensitive dentine, it can be accomplished either by a total anæsthesia of the tooth, or by a local anæsthesia of the ends of the fibrils. The general anæsthesia is best accomplished by the application of cold produced by chloride of ethyl. Care should be taken not to apply the cold where there is an inflamed pulp, as great pain will be produced. But it can be applied very successfully in cases of sensitive dentine and in slight grades of hyperæsthesia which react up to 22° C., as described above. If used in cavities which react to a temperature above 22° C., it is a great mistake.

Where cold cannot be used, resort must be had to other means affecting a more local area. Of these the author mentions cocaine and chinose. The latter drug acts by favoring the diffusion of the cocaine. A stream of warm carbonic acid gas is also recommended. Carbonic acid gas is said to lower the vitality of tissues with which it comes in contact and to make them less capable of transmitting irritations. The author's latest method is to produce carbonic acid gas in the cavity in a nascent condition, and use in combination some alkaloid, preferably cocainum nitricum.

William H. Potter.

